# Unexpected Cataract Formation Following Valproate Treatment: A Case Report

**DOI:** 10.7759/cureus.63093

**Published:** 2024-06-25

**Authors:** Nayan Sinha, Pradeep S Patil, Aditi Ananta, Sneha B Suresh, Navaneetha Unni

**Affiliations:** 1 Psychiatry, Jawaharlal Nehru Medical College, Datta Meghe Institute of Higher Education & Research, Wardha, IND; 2 Ophthalmology, Jawaharlal Nehru Medical College, Datta Meghe Institute of Higher Education & Research, Nagpur, IND

**Keywords:** valproate induced cataract, valproic acid, bipolar affective disorder, cataract, valproate toxicity

## Abstract

Valproic acid (VPA), or sodium valproate, is a frequently prescribed medication for many psychiatric conditions, notably for the management of bipolar affective disorder. While its common side effects are well known and thoroughly documented in medical literature, the occurrence of cataracts as a side effect is exceedingly rare. There is evidence of cataract formation with long-term use of VPA in a few studies. Recognizing this potential adverse effect is crucial. It is important to recommend that patients undergo regular eye examinations if they experience any visual disturbances or as a preventative measure to ensure effective management. This case report examines the unusual occurrence of cataract development associated with valproate use.

## Introduction

Valproic acid (VPA) is an anticonvulsant and a commonly used medication for psychiatric and neurological conditions. It is utilized for treating epilepsy across a range of seizure types, managing bipolar disorder, and preventing migraines [1]. Common side effects of VPA encompass hematological, dermatological, neurological, and gastrointestinal symptoms. These typically manifest at the onset of VPA treatment or after dosage adjustments. However, they often diminish over time or with appropriate dose modifications [2].

There are some severe adverse reactions that can happen after VPA treatment. Hematological complications include thrombocytopenia, leukopenia, bone marrow suppression, aplastic anemia, and coagulation disorders. Life-threatening dermatological reactions like toxic epidermal necrolysis, Stevens-Johnson syndrome, and anaphylaxis have been reported. Other serious adverse events include hepatotoxicity, neuropsychiatric disturbances (hallucinations, suicidal ideation, and psychosis), and electrolyte abnormalities such as hyponatremia, pancreatitis, polycystic ovarian syndrome, cerebral pseudoatrophy, encephalopathy, and coma. While these adverse effects are rare, they can potentially be life-threatening [3]. It is not recommended for use during pregnancy due to its teratogenic effects, which can result in a condition known as fetal valproate syndrome. This syndrome is characterized by various facial features, such as an infraorbital groove, epicanthic folds, sparse eyebrows, a shallow nasal bridge, a short nose with trigonocephaly, anteverted nares, and a tall forehead. Additionally, previous literature has reported a high occurrence of early-onset myopia in cases of fetal valproate syndrome [4,5].

Visual abnormalities linked to VPA include diplopia (double vision), blurred vision, and nystagmus (involuntary eye movements) [6]. While relatively uncommon, some articles have also demonstrated an association between cataracts and the use of VPA [7,8]. Therefore, the main aim of this case report is to provide evidence of cataract formation with long-term use of VPA or sodium valproate.

## Case presentation

We present the case of a 27-year-old unmarried male, educated until the first year of engineering college, from a low socioeconomic nuclear family with a well-adjusted pre-morbid personality. He exhibited symptoms including overtalkativeness, overfamiliarity, grandiosity, decreased need for sleep, and increased activity over the past month. Following admission and a detailed evaluation, he was diagnosed with bipolar affective disorder, a current episode of mania, according to the International Classification of Diseases-11 (ICD-11) [9]. This hospitalization marked his first admission to our hospital.

The patient had a history of bipolar affective disorder, experiencing three documented manic episodes over the past seven years in 2017, 2019, and 2022. He had been under the care of another psychiatrist and had been diagnosed with this condition. His maintenance treatment regimen included sodium valproate tablets, with a total daily dose of 1,500 mg divided into 500 mg in the morning and 1,000 mg at night, which he had been taking for seven years. There was intermittent noncompliance for approximately 15 days, occurring only during his manic episodes. During previous manic episodes, he was prescribed haloperidol tablets (10 mg) once daily at bedtime, which were tapered and discontinued once the manic symptoms resolved. He had no history of substance abuse or exposure to smoke, and there were no known medical comorbidities.

**Figure 1:**
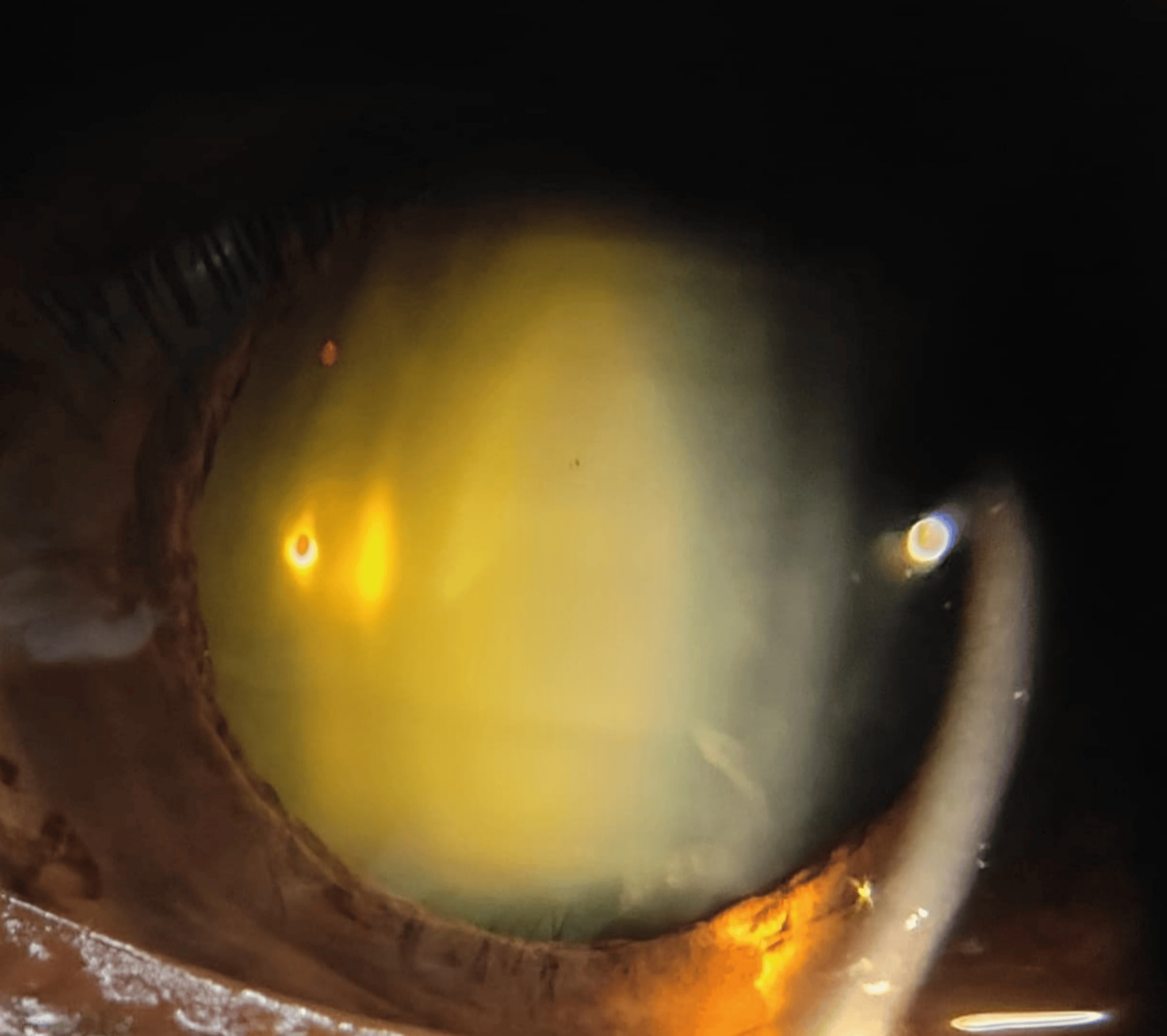
Posterior subcapsular cataract of the right eye

**Figure 2:**
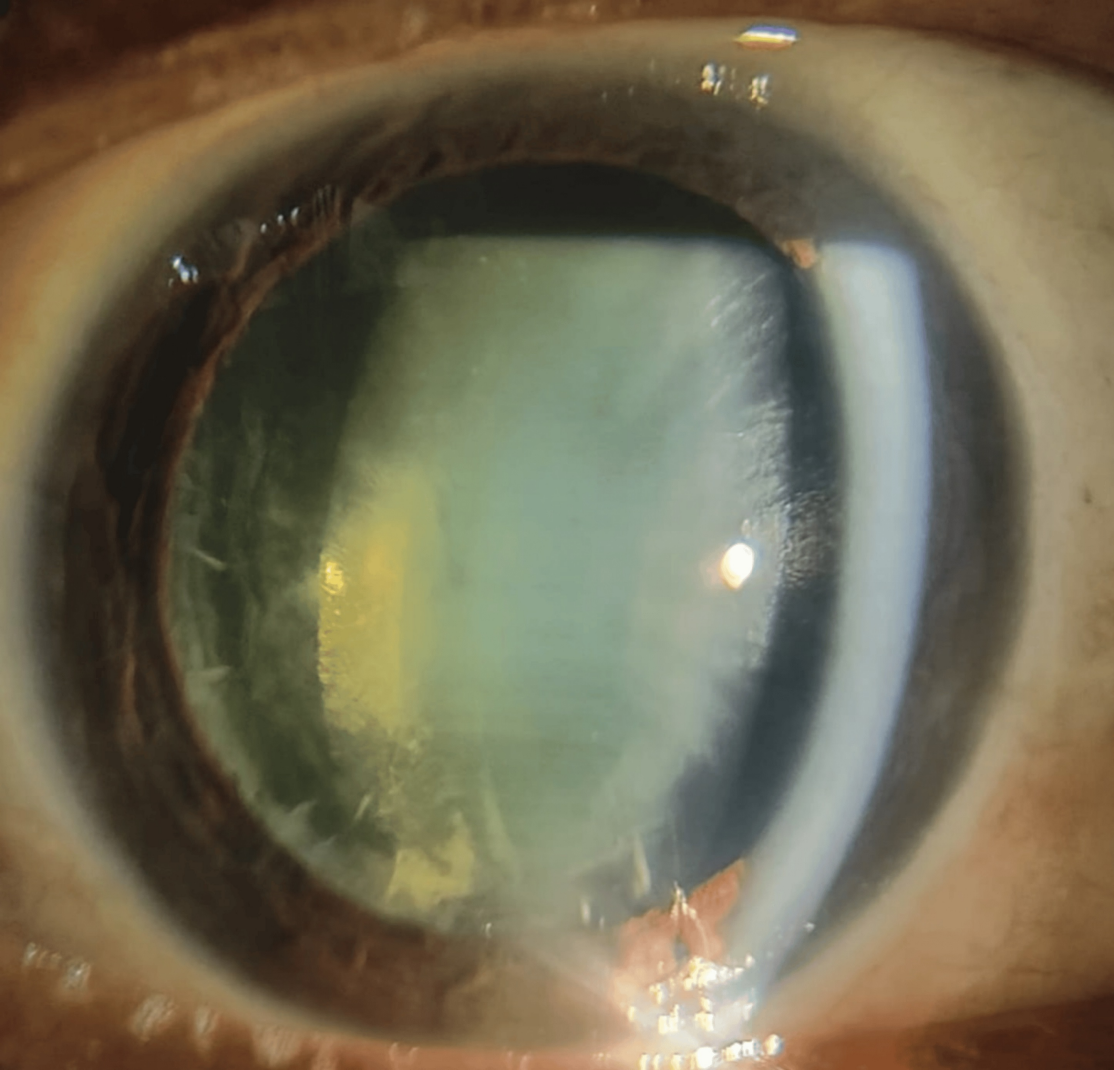
Posterior subcapsular cataract of the left eye

In his medical history, it was revealed that the patient had complained of painless, gradually diminishing vision over the past year, which had progressively worsened. He was admitted to the ophthalmology department for an evaluation of his vision deficits. His visual acuity was measured at 1/60 in each eye. Intraocular pressure was 16 mm Hg in both eyes, assessed using a noncontact tonometer. Examination with a slit-lamp bi-microscope revealed cataractous changes in both eyes, graded as posterior subcapsular cataracts (Figure 1, Figure 2).

The fundus examination of both eyes was hazy, the disc appeared normal, and other details were not clearly visible due to dense posterior subcapsular cataracts. According to the patient’s medical records, hematological and serological results, including a complete blood count, liver function test, renal function test, serum vitamin B12, serum vitamin D, lipid profile, fasting and post-prandial blood sugar, and serum calcium, were within normal limits. There was no history of asthma, hypertension, diabetes mellitus, eye trauma, or any other vision deficits, including congenital cataracts. There was no indication, including any positive family history, for cataracts. The patient underwent bilateral cataract extractions using phacoemulsification and posterior chamber intraocular lens implantations. Following the procedure, his visual acuity improved to 6/6 in both eyes.

During this period, he had stopped taking his medications, resulting in a relapse of symptoms, and he was brought to the psychiatry department. We initiated treatment with an olanzapine tablet (10 mg) once a night. His manic symptoms subsided, and he was maintained on the same medication thereafter.

## Discussion

A cataract is a condition where the lens of the eye, usually transparent, becomes cloudy or opaque, hindering the passage of light to the retina. This condition, which can lead to blindness, can impact individuals across various age groups but is more common among older adults [10]. Its prevalence tends to be higher among females compared to males in both developed and developing nations. Additionally, in developing countries, it tends to manifest at an earlier age. A notably high prevalence rate of cataracts has been noted in population-based studies conducted in India.

The clouding or opacity of the lens and/or its capsule occurring before the age of 40, after excluding all other known causes of cataracts, is termed a presenile cataract [11]. Cataracts are less frequently observed in young adults, typically arising because of factors such as eye injury, inflammation within the eye, uncontrolled hypertension and diabetes, hypoparathyroidism, metabolic disorders present from birth, long-term low calcium levels, and extended use of steroids. Additionally, conditions like high myopia, persistent dehydration, allergic dermatitis arising after eye surgery for other conditions, ongoing uveitis, tobacco use, extended exposure to sunlight, and alcohol consumption are noted as potential contributors to cataract development in younger individuals [8,12].

Previous studies conducted in India have linked several factors to the development of presenile cataracts, including prolonged exposure to smoke, dyslipidemia, hypercholesterolemia, low levels of vitamin D, hypertriglyceridemia, and exposure to fuels, in addition to the factors mentioned above [11,13].

In recent times, numerous studies have highlighted the potential for certain widely prescribed antipsychotic and antidepressant medications to contribute to cataract formation. However, there has been relatively limited research into the cataract-inducing effects of commonly used mood stabilizers. Based on literature findings, long-term use of lithium, either alone or in combination with other mood stabilizers, as well as the combination of VPA with other mood stabilizers, for durations exceeding two years, appears to elevate the risk of cataract development among individuals diagnosed with bipolar disorder and schizophrenia [14]. There is activation of the chronic unfolded protein response by sodium valproate. This results in the epigenetic modification of the Kelch-like enoyl-CoA Hydratase-associated protein 1 ne and proteasomal degradation, which in turn suppresses the nuclear factor-erythroid-2-related factor 2-dependent stress and antioxidant protection. This leads to lens oxidation and cataract formation [7,15].

In this scenario, the progression of cataracts following prolonged sodium valproate therapy for bipolar affective disorder, coupled with the absence of any other apparent cause, strongly indicates that sodium valproate may have contributed to the development of the cataract. Our patient had no risk factors, family history of cataracts, or any indications of developing cataracts at a young age. The Naranjo adverse drug reaction probability scale was applied to assess the patient, revealing a score of 7, suggesting that valproate is likely the cause of the patient’s cataract [16]. After ruling out all the causes, it was concluded that his condition was drug induced. The patient was maintained on antipsychotics alone due to the risk of cataract formation with mood stabilizers [14].

## Conclusions

This case highlights the potential risk of cataract development associated with prolonged sodium valproate therapy. Such rare adverse effects tend to be underrecognized and underreported. Psychiatrists need to be mindful of the potential cataract-inducing effects of sodium valproate, frequently prescribed for epileptic conditions and bipolar affective disorder, particularly with prolonged use. It is essential to conduct regular ophthalmic assessments throughout extended sodium valproate therapy to mitigate the risk of cataract development and its associated complications.
